# Data on optimization of expression and purification of AIMP2-DX2 protein in *Escherichia coli*

**DOI:** 10.1016/j.dib.2017.03.011

**Published:** 2017-03-10

**Authors:** Roshan Jha, Hye Young Cho, Ameeq Ul Mushtaq, Kiho Lee, Dae Gyu Kim, Sunghoon Kim, Young Ho Jeon

**Affiliations:** a College of Pharmacy, Korea University, 2511 Sejong-ro, Sejong 30019, Republic of Korea; b Medicinal Bioconvergence Research Center, Seoul National University, Seoul 08826, Republic of Korea; c Research Institute of Pharmaceutical Sciences, College of Pharmacy, Seoul National University, Seoul 08826, Republic of Korea

**Keywords:** AIMP2, Aminoacyl-tRNA synthetase interacting multifunctional protein 2, AIMP2-DX2 or DX2, splicing variant of AIMP2 lacking exon 2, DTT, dithiorietol, IPTG, isopropyl β-D-1-thiogalactopyranoside, SDS-PAGE, Sodium dodecyl sulfate polyacrylamide gel electrophoresis, SUMO, Small Ubiquitin-like modifier, AIMP2, AIMP2-DX2, Expression and solubility, Protein stabilizers, SUMO-tag

## Abstract

AIMP2-DX2 is a splicing variant of AIMP2 protein which has been implicated in human lung cancer and chemoresistance of ovarian cancer (J.W. Choi, D.G. Kim, A.E. Lee, H.R. Kim, J.Y. Lee, N.H. Kwon, et al., 2011; J.W. Choi, J.W. Lee, J.K. Kim, H.K. Jeon, J.J. Choi, D.G. Kim, et al., 2012) [1,2]. We have shown, here, the data for the expression of AIMP2-DX2 protein in *Escherichia coli* and optimization of the critical steps in purification of AIMP2-DX2. The data described here has been successfully used to get a maximum yield of highly pure AIMP2-DX2 for subsequent characterization of its biophysical property in: “Purification and biophysical characterization of the AIMP2-DX2 protein” (R. Jha, H.Y. Cho, A. Ul Mushtaq, K. Lee, D.G. Kim, S. Kim, et al., 2017) [3].

**Specifications Table**

**Table:** 

Subject area	*Biochemistry, Structural Biology*
More specific subject area	*Protein Expression and purification*
Type of data	*Table and figures*
How data was acquired	*Sodium dodecyl sulfate polyacrylamide gel electrophoresis (SDS-PAGE)*
Data format	*Raw, analyzed*
Experimental factors	*Common laboratory practices for production of recombinant proteins from E.Coli, Common protocol for SDS-PAGE analysis*
Experimental features	*Optimization of protein expression in E.Coli, optimization of protein purification, Yield of protein*
Data source location	*Korea University, Sejong, South Korea*
Data accessibility	*Data is within this article*

**Value of the data**

• The data includes a process for screening the expression system and optimizing the purification steps for human AIMP2-DX2 protein. This data provides an efficient method to get a high yield of AIMP2-DX2 protein.
• The data on SUMO-tag cleavage shows that the protease activity of SUMO-proteases is efficient even at a salt concentration of 500 mM NaCl.
• The data on screening the optimal condition for protein purification and stabilization shows that EDTA can be a good additive to prevent aggregation of protein by chelating residual nickel and other metallic impurities from Nickel affinity chromatography.

## 1 Data

**Fig. 1:**
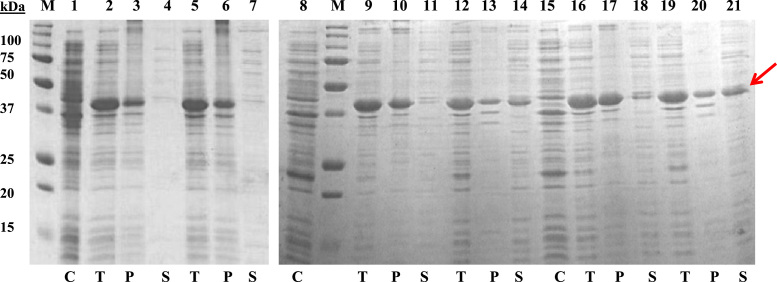
Screening of solubility of 6His-SUMO-DX2 protein in different *E. coli* cell lines by SDS-PAGE. M-protein size marker; C-no IPTG induction, T-total lysate, P-pellet of lysate and S-supernatant of lysate. Lanes 1–7 test of protein solubility in BL21(DE3) cell line, lanes 2–4 is for induction at 37 °C and lanes 5–7 is for induction at 18 °C. Lanes 8–14 test of protein solubility in BL21-CodonPlus(DE3)-RIL cell line, lanes 9–11 is for induction at 37 °C and lanes 12–14 is for induction at 18 °C. Lanes 15–21 test of protein solubility in BL21-CodonPlus(DE3)-RIPL cell line, lanes 16–18 is for induction at 37 °C and lanes 19–21 is for induction at 18 °C. Red arrow denotes the AIMP2-DX2 band which was used for purification in this study.(For interpretation of the references to color in this figure legend, the reader is referred to the web version of this article.).

Fig. 1 shows the SDS-PAGE image of the expression and the solubility trial of the AIMP2-DX2 protein with a SUMO-tag in various *E. coli* cell lines. The *E. coli* cells were expressed at 37 °C and 18 °C with 1 mM IPTG. These cells were harvested, lysed and separated as pellet and supernatant before subjecting to SDS-PAGE. The variation in the solubility of SUMO-DX2 at different temperature and cell lines is easily comparable with relative protein band intensity.

**Fig. 2:**
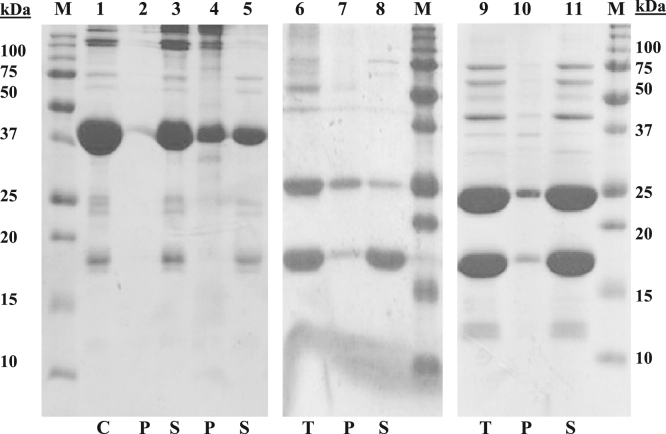
Optimization of dialysis and SUMO tag cleavage of 6His-SUMO-DX2 protein. M-protein size marker; C - control, T - total protein, P - precipitated protein and S - soluble protein. Lanes 1–5 is SDS-PAGE analysis after dialysis of 6His-SUMO-DX2 (42 kDa); lane 1 is protein sample before dialysis; lane 2 is precipitated fraction and lane 3 is soluble fraction after dialysis against buffer 50 mM Tris–HCl pH 7.4, 500 mM NaCl, 1 mM DTT. Note that elution fraction was mixed with EDTA at a final concentration of 10 mM before dialysis; lane 4 is precipitated fraction and lane 5 is soluble fraction after dialysis against buffer 50 mM Tris–HCl pH 7.4, 100 mM NaCl, 1 mM DTT. Lanes 6–11 is SDS-PAGE analysis after cleavage of SUMO-tag showing free DX2 (27.8 kDa) and 6His-SUMO (14 kDa); lane 6 is total protein, lane 7 is precipitated fraction and lane 8 is soluble fraction of protein after tag cleavage in buffer 50 mM Tris–Cl pH 7.4, 100 mM NaCl, 1 mM DTT; lane 9 is total protein, lane 10 is precipitated fraction and lane 11 is soluble fraction of protein after tag cleavage in buffer 50 mM Tris–HCl pH 7.4, 500 mM NaCl, 1 mM DTT.

Fig. 2 shows the SDS-PAGE image comparing the improvement in the stability of the SUMO-tagged AIMP2-DX2 between the optimized and unoptimized buffer conditions. The protective effect of 500 mM NaCl and 10 mM EDTA has been shown here for two critical processes, dialysis and SUMO-tag cleavage, during the purification of AIMP2-DX2.

## 2 Experimental design, materials and methods

### 2.1 Cloning and expression of protein

Full length human AIMP2 isoform C (hereafter referred as AIMP2-DX2 or DX2) protein was amplified from a PGEX-4T-1 plasmid containing human DX2, reported previously by Choi and Lee et al. [1], [2]. The splicing variant of AIMP2 (NCBI nucleotide accession number NM_006303 and Genbank code U24169) lacking exon2 was generated as described by Choi et al. [1]. To get a high yield of soluble DX2 protein, we designed a cloning strategy to express DX2 with a SUMO protein tag. For this purpose, the yeast SUMO protein (Smt3; Genbank ID BK006938) was cloned into the pET-28a plasmid (Novagen, Germany) between Nhe I and BamH I restriction sites with procedures similar to that reported by Malakhov et al. [4]. Next, the amplified DX2 gene fragment was inserted into this modified pET-28a-SUMO plasmid between restriction sites BamH I and Xho I. The *Taq* DNA polymerase and all the RE enzymes used were obtained from Enzynomics (South Korea). Standard cloning procedures were followed and the success of the cloning strategy was assessed by DNA sequencing.

The plasmid construct, thus obtained, would express the DX2 protein with a hexahistidine tag followed by a SUMO tag at its N-terminal (for convenience designated as 6His-SUMO-DX2 protein). For screening the best expression system, the 6His-SUMO-DX2 plasmid construct was transformed into four *E. coli* cell lines, namely, BL21(DE3) (Novagen, Germany), BL21-CodonPlus(DE3)-RIL (Stratagene, USA) and BL21-CodonPlus(DE3)-RIPL (Stratagene, USA). All the *E. coli* cells were expressed at temperatures 37 °C and 18 °C with 1 mM IPTG. The expression and the solubility of the 6His-SUMO-DX2 protein were analyzed by SDS-PAGE and the relative band intensity was compared to determine the best *E. coli* expression system.

### 2.2 Optimization of purification process

DX2 expressed in *Escherichia coli* BL21-CodonPlus(DE3)-RIPL (Stratagene, USA) at 18 °C was cultured, harvested and then lysed by ultrasonication. To purify DX2, this lysate was subjected to nickel affinity chromatography followed by dialysis, tag cleavage and second nickel affinity chromatography. Size exclusion chromatography was used to further separate the oligomer fraction of AIMP2-DX2 (see reference [1] for details). During the dialysis of the elution fraction from nickel affinity chromatography to standard buffer for SUMO-tag cleavage (50 mM Tris–HCl pH 7.4, 150 mM NaCl, 1 mM DTT) and the subsequent tag cleavage, precipitation of SUMO-DX2 was severe with loss of about 50–60% of protein. To optimize this step of purification, 500 ml of buffers with several different additives were prepared. Then, the stability of 1–2 ml of elution fractions on dialyzing to these buffers and subsequent tag cleavage was analyzed with SDS-PAGE. Osmolytes (glycerol, mannitol, betaine and trehalose), the salts of the Hofmeister series (MgSO_4_, MgCl_2_, Na_2_SO_4_, NaCl and KCl) and other common additives like EDTA, DTT and mild detergents were checked for their protective action [5], [6]. From the initial screening process, NaCl and EDTA showed the most protective effect. Both NaCl and EDTA were further screened for their protective effect at various concentrations. After several rounds of screening, the most optimal method was to mix the elution fractions from nickel affinity chromatography with EDTA at concentration of 10 mM before extensive dialysis to buffer solution 50 mM Tris–HCl pH 7.4, 1 mM DTT and 500 mM NaCl. This buffer condition was also most suitable for SUMO-tag cleavage. It should be noted that the absence of the EDTA in the buffer during tag cleavage had no effect on the stability of the protein.

These data has been successfully used to get a maximum yield (10 mg/L) of highly pure (> 95%) AIMP2-DX2 for subsequent characterization of its biophysical property in: “Purification and biophysical characterization of the AIMP2-DX2 protein” (R. Jha, H.Y. Cho, A. Ul Mushtaq, K. Lee, D.G. Kim, S. Kim, et al., 2017) [3].
